# Blunt thoracic aortic injury – concepts and management

**DOI:** 10.1186/s13019-020-01101-6

**Published:** 2020-04-19

**Authors:** Nicolas J. Mouawad, Joseph Paulisin, Stephen Hofmeister, Matthew B. Thomas

**Affiliations:** McLaren Bay Heart & Vascular, McLaren Bay Region, 1900 Columbus Avenue, 4th Floor, South Tower, Bay City, MI 48708 USA

**Keywords:** Blunt thoracic trauma, Aorta, Aortic injury, TEVAR, Trauma

## Abstract

**Background:**

Blunt thoracic aortic injury, a life-threatening concern, remains the second most common cause of mortality among all non-penetrating traumatic injuries, second only to intracranial hemorrhage. Kinetic forces from the rapid deceleration are the impetus for the injury mechanism and are graded accordingly. Given the prevalence of trauma as a public health problem, contemporary management considerations are important.

**Main body:**

Blunt thoracic aortic injury may be fatal if not diagnosed and treated expeditiously. Endovascular options allow safe and effective management of these dangerous injuries. This paper describes the overview of blunt thoracic aortic trauma, the epidemiology, presentation, diagnosis, and treatment options with a focus on endovascular management.

**Conclusion:**

Blunt thoracic aortic injury requires a high index of suspicion based on mechanism of injury in the trauma population. Endovascular options have become the mainstay of blunt thoracic aortic injury treatment whenever feasible with satisfactory results and long-term outcomes.

## Background

Trauma remains the most common cause of death in young adults. Head injury is most prevalent followed by blunt thoracic aortic injury (BTAI), which has been demonstrated by multiple autopsy studies [1–4]. In the majority of cases, high energy blunt trauma with a rapid deceleration is present. Motor vehicle accidents, particularly head on collisions, account for 81% of the cases [5]. Motorcycle crashes, falls from significant height, crush injuries, pedestrian vs motor vehicle accidents and aircraft injuries account for the other 19% of cases. Up to 80% of patients presenting with BTAI die before hospitalization, and in the remaining survivors, in-hospital mortality is as high as 46% [3, 4, 6]. While this is a potentially lethal injury, it is rare and accounts for 1.5% of thoracic trauma [1, 3, 4].

Rapid deceleration is the universal mechanism of this injury. Most commonly, there are multiple other life-threatening injuries present with less than 20% having this as an isolated injury making the diagnosis and initial next steps challenging [2, 4]. BTAI is defined as a tear in the aorta that is a result of a combination of shear and stretch forces [7], rapid deceleration, increased intravascular pressure [8] and compression of the aorta between the anterior chest wall and vertebrae [9]. Injury can occur along the entire length of the aorta, essentially from the ascending aorta to the iliac bifurcation, although the injury typically occurs areas of aortic tethering, notably the aortic isthmus. There exist three major theories that account for the mechanism of injury (it is likely that they collectively explain the mechanism and not one explanation is correct by itself): primarily, the aortic isthmus is a transition zone from the unfixed aortic arch to the fixed descending aorta – this area is predisposed to go in opposite vectors during a rapid deceleration and can lead to a tear in the intima [10, 11]; secondly, weaker tensile strength exists in the tissue of the aortic isthmus making it intrinsically more vulnerable to injury [10, 12]; and thirdly, a compression point between the anterior chest’s osseous structures and the spine acts in a way that pinches the aorta during a deceleration trauma [10, 13]. Porcine models identified an initial tear in the intima and media first from these forces causing a dissection [14]. Blood fills the dissection plane which can ultimately penetrate the adventitia causing a pseudoaneurysm and/or free rupture. The advancement from an intimal tear to free rupture happens over a variable amount of time. Increased blood pressure and afterload clearly decrease the interval in which this happens. Also, rapid overzealous fluid resuscitation and transfusions may hasten this advancement.

## Main body

A high index of suspicion is mandatory based on pre-clinical presentation and mechanism of action. The management and the process of diagnosis should follow ATLS protocol and prioritize primary, secondary and tertiary surveys with the appropriate diagnostic tests being performed based on the patient’s clinical status and the facility’s capabilities. Appropriate resuscitation tactics and triaging to definitive care facilities is necessary as many level 2 and 3 trauma centers do not have the capabilities to care for these injuries definitively, and early transfer if necessary should be performed.

Early diagnosis is paramount as many patients with blunt thoracic aortic injury die within 24 h if unmanaged. Due to trauma patients commonly being mentally incapacitated for a variety of reasons (shock/hypoperfusion, intoxication or being intubated and sedated), chief complaints driving this diagnosis may not be present. Chest related complaints are not sensitive or specific to blunt thoracic aortic injury either but should raise more suspicion. Blunt thoracic aortic trauma is associated with other major entities of chest trauma, including, but not limited to, sternal fracture, 1st/2nd rib fractures, clavicle and/or scapular fractures, pneumothoraces, hemothoraces, flail chest, pulmonary contusions, diaphragm injury, tracheobronchial disruption and esophageal injuries; these should raise suspicion for BTAI. Physical exam findings also have low sensitivity and specificity for this injury. Contusion and abrasions of the chest wall may increase suspicion but once again are not sensitive or specific. Upper extremity hypertension may be uncommonly present due to a pseudo-coarctation from the dissection. This is especially uncommon in the critically ill polytrauma patient presenting in shock.

### Imaging

During the primary survey, plain anteroposterior chest x-ray is almost universally utilized as an adjunct test. There are many chest x ray findings that raise suspicion for blunt thoracic aortic injury, but the sensitivity and specificity is not high enough to reliably diagnose or rule out blunt thoracic aortic injury. Widened mediastinum is perhaps the easiest and most common to recognize. Other findings include but are not limited to: loss of aortic knob, apical capping from blood in the apex, large left pleural effusion (hemothorax), displacement of left mainstem bronchus, OG/NG tube to the right, deviation of the trachea or right mainstem bronchus and widening of the paravertebral stripe. FAST scan ultrasounds are almost ubiquitous in emergency departments and this has been heightened by the adoption of bedside ultrasound imaging to the practice of emergency medicine. Free rupture of the aorta can lead to a left hemothorax and pericardial effusion leading to tamponade, both of which can be seen on FAST. However, these findings on bedside ultrasound also have a low sensitivity/specificity and would mean late signs of the disease process if present.

A major shift in the last few decades with respect to diagnosis of BTAI is the regular use of computed tomographic angiography (CTA) which has become by far the imaging modality of choice [15]; in fact, the increased use of CTA has potentially led to a rise in the incidence of BTAI due to previously undetected minimal aortic injuries with the use of older less sensitive imaging methods [16]. Transesophageal echocardiography (TEE) is also a good option in the unstable polytrauma patient that cannot be transported to radiology for CTA. An intimal flap, aortic valve regurgitation, an intramural hematoma or signs of a free rupture can be identified and help expedite treatment and medical management where appropriate. If the unstable patient is currently in the operating room for control of hemorrhagic shock at another site, the TEE results can assist in guiding resuscitation approaches (i.e. permissive hypotension) and identifying this injury. This test however is operator dependent.

### Medical management/resuscitation phase

Initial management involves primary and secondary surveys with adjunctive tests that are appropriate for a patient’s clinical status. Sufficient monitoring and large bore IVs should be obtained. This assessment includes the treatment and identification of hemorrhagic shock. However, once blunt thoracic aortic injury is suspected or diagnosed, permissive hypotension and reasonable volume expansion should be practiced, preventing expanding the dissection to rupture or end organ damage. Heart rate and blood pressure control can slow the expansion of the blunt thoracic injury. Goal systolic blood pressure and heart rate are 100 mm hg and less than 100 bpm respectively [10]. In a center without definitive care, these parameters can be used to assist in transferring a stable patient or a bleeding patient with controlled extra-aortic sources of hemorrhage to a definitive care center.

Esmolol is the medication of choice for blunt thoracic aortic injury secondary to its rapid onset and short half-life, making it easy to wear off if hemorrhagic and other causes of shock should arise [10]. Diltiazem, nitroglycerin and nitroprusside can also be used in conjunction with or alternative to esmolol, although understanding their pharmacokinetics and side effects is imperative. Importantly, pharmacologic control of blood pressure and heart rate dramatically reduce the risk of rupture to < 2% [17, 18].

### Grading and management

The current standard of care for grading these injuries was proposed in 2009 and has been adopted by the Society for Vascular Surgery clinical practice guidelines for the management of BTAI [19, 20]. Injuries are assigned one of 4 grades based on CTA imaging: grade 1 (intimal tear), grade II (intramural hematoma), grade III (pseudoaneurysm) and grade IV (rupture). Currently, the recommendation is to proceed with surgical repair of Grade II-IV injuries [20]. For grade I injuries, it is well established that no intervention is necessary as these tend to resolve on their own with conservative management. Grade II injures do fall into a “gray zone” between medical management and operative intervention although more recent studies do document that nonoperative is safe with close follow up [4]. If low grades are being medically managed with blood pressure and heart rate control but subsequent imaging demonstrates advancement of grading, delayed repair is indicated. Grade III injuries are able to repaired in a delayed fashion using endovascular endografting while more pressing traumatic injuries are managed first following initial stabilization. Unfortunately, most grade IV patients do not make it to the hospital in time, and if they do, they are in extremis and require immediate intervention. In the polytrauma patient, those with grade II or higher without active bleeding from the aorta, delaying the repair and allowing time for the stress of the trauma to subside and allow more resuscitation may allow a better prognosis. If the patient is unstable or bleeding from a high-grade injury, immediate repair is the only choice. Delayed repairs do better than immediate repairs, but this is likely due to immediate repairs being in more critically ill patients.

In those in which repair is indicated, either an open or endovascular approach is performed. Given the location of injury in 50–70% of cases [17], traditional surgical repair typically involves a high posterolateral thoracotomy with or without cardiopulmonary bypass and significant blood loss, which can negatively impact other organ systems including prolonged ventilation and neurologic morbidity such as stroke and paraplegia [21–24]. Respiratory concerns from chest and lung injuries are compounded with open thoracotomy particularly if lung contusions are present making single-lung ventilation difficult. Adequate decubitus positioning may be risky particularly with patients with spinal fractures [25]. Historically, open repair of traumatic aortic injuries have been associated with a 16% paraplegia rate and a 28% mortality rate [26–28]. Open repairs are typically performed with an interposition graft being placed where the injury formed. Primary repair is very rare but is sometimes possible. The clamp and sew technique was the most common method of treatment for a long time [29]. However, due to high rates of clamp related ischemia of distal organs and the spinal cord, techniques were developed using a bypass circuit to actively perfuse the distal aorta past the injury repair site such left atrial/right pulmonary vein to femoral artery bypass or the femoral vein to femoral artery bypass. Both require heparin which may be contraindicated if head trauma or active bleeding present [29]. These techniques of active distal aorta perfusion have shown significant decreases in immediate morbidity and mortality but not enough to compete with the results of endovascular stent grafting.

### Endovascular approach

Thoracic stent grafts were originally made for treating aneurysmal disease. In 1994, Dake and colleagues reported preliminary results indicating that endovascular stent graft repair is safe in highly selected patients with descending thoracic aortic aneurysms [30]. Semba and colleagues in 1997 showed that stent graft repair is technically possible in acute descending thoracic aortic rupture [31]. Over the last 2 decades, thoracic endovascular aortic repair (TEVAR) has revolutionized the intervention for descending thoracic aortic pathology, and several studies have confirmed this therapeutic approach for BTAI [32–40]. The prospective Second American Association for the Surgery of Trauma (AAST) trial evaluated the impact of TEVAR for BTAI across major trauma centers in the United States. The adoption and use of TEVAR increased dramatically from 0 to 65% between the first AAST investigation in 1997 and the second in 2007 [35]. The Second AAST’s comparative analysis identified a 16% mortality rate in open repair as opposed to 9% in the TEVAR group (*P* = .001). Interestingly, despite a high clamp-and-sew rate in the open group (16%), the incidence of procedure-related spinal cord injury was a low 2.9% in the open group versus 0.8% in the TEVAR group (*P* = .28) [35]. Medicare cites that an approximately 7 time increase in the amount of TEVAR for blunt thoracic aortic injury has occurred between 2004 and 2007 in other studies, from 11 to 76% [41]. This continues to increase and is believed to be even higher now.

Endovascular candidacy primarily depends on landing zone for the aorta 2 cm proximal to the injury and 10 cm distal to the injury. 3D reconstruction with CTA is recommended for appropriate diameter measurements at these two sites. If not available, intravascular ultrasound should be used to ensure that the graft chosen is not over or under sized too much. Access vessel requirements depend on the device but the typical limiting factor is iliac arteries of 7 mm or less in diameter. An iliac conduit may be necessary but should be reconsidered if a pelvic hematoma or fracture is present as this could be disrupted and lead to massive bleeding. If the ascending aorta or more proximal arch is involved the patient should not be treated endovascularly with current technology. However, new age branched and fenestrated grafts are being developed and may be considered in patients without valvular insufficiency.

At the 2 cm proximal landing zone, the diameter of the aorta will determine candidacy and size of graft. These values are device specific and are dependent on contemporary supply chain and local institution value analysis. Arch anatomical variations need to always be kept in mind to avoid covering the left common carotid artery. Ten to 20 % oversizing is recommended but with a tendency to stay on the lower side of that range. It has been noted that unlike aneurysmal disease oversizing is not as critical. The typically smaller diameter and tortuous thoracic aorta of young trauma patients make these cases difficult. Endografts have been initially built for aneurysmal disease therefore it may be necessary to use aortic cuffs or even conduits if the delivery system is too short.

Spinal drains in the setting of blunt thoracic aorta injury are typically not necessary although should be considered in patients with previously covered abdominal aorta or occluded iliac vessels because needed collaterals to the spinal cord will be limited and may not allow adequate compensatory blood flow. Importantly, {SPP (spinal perfusion pressure) = MAP (Mean Arterial Pressure) – SAP (subarachnoid pressure)}: from the equation, draining CSF from the spinal drain lowers SAP and hence increases spinal perfusion pressure. This allows for collateral flow to compensate the best it can for ischemia caused by thoracic aortic coverage over spinal arteries. Increasing MAP can be performed with vasopressors which will ultimately increase the SPP.

A multidisciplinary approach to this intervention should be universal. Preoperative planning with experienced clinicians and the consultation of an experienced device manufacturer representative is critical for success. The appropriate combination of vascular specialists with team members capable of open repair available can make for success. The majority of endovascular complications can be dealt with by endovascular means and not requiring open conversion.

It is important to note that delayed repair may improve survival where immediate operation for aortic surgery is prohibitive due to the polytrauma that would carry a high mortality risk [42, 43]. However, there has been a risk of delayed rupture in the unrepaired thoracic aortic transection estimated between 2 to 5% [44–46]. Randomized control studies between open versus endovascular repair of blunt thoracic aortic trauma will never be performed and would likely be stopped early in the study due to the higher immediate mortality and morbidity of open repair. Open repair is still sometimes needed when a patient’s injury has anatomical features that are not suitable for endovascular approach. These anatomical features include arch type, tortuosity, diameter of proximal landing zone and iliac/access vessel sizes and features. While the open repairs are more durable and require far less re-intervention, their early morbidity/mortality is significantly higher [32]. Open repair should be looked at as complementary rather than competing and still a necessary procedure to have available in certain circumstances [6].

**Fig. 1:**
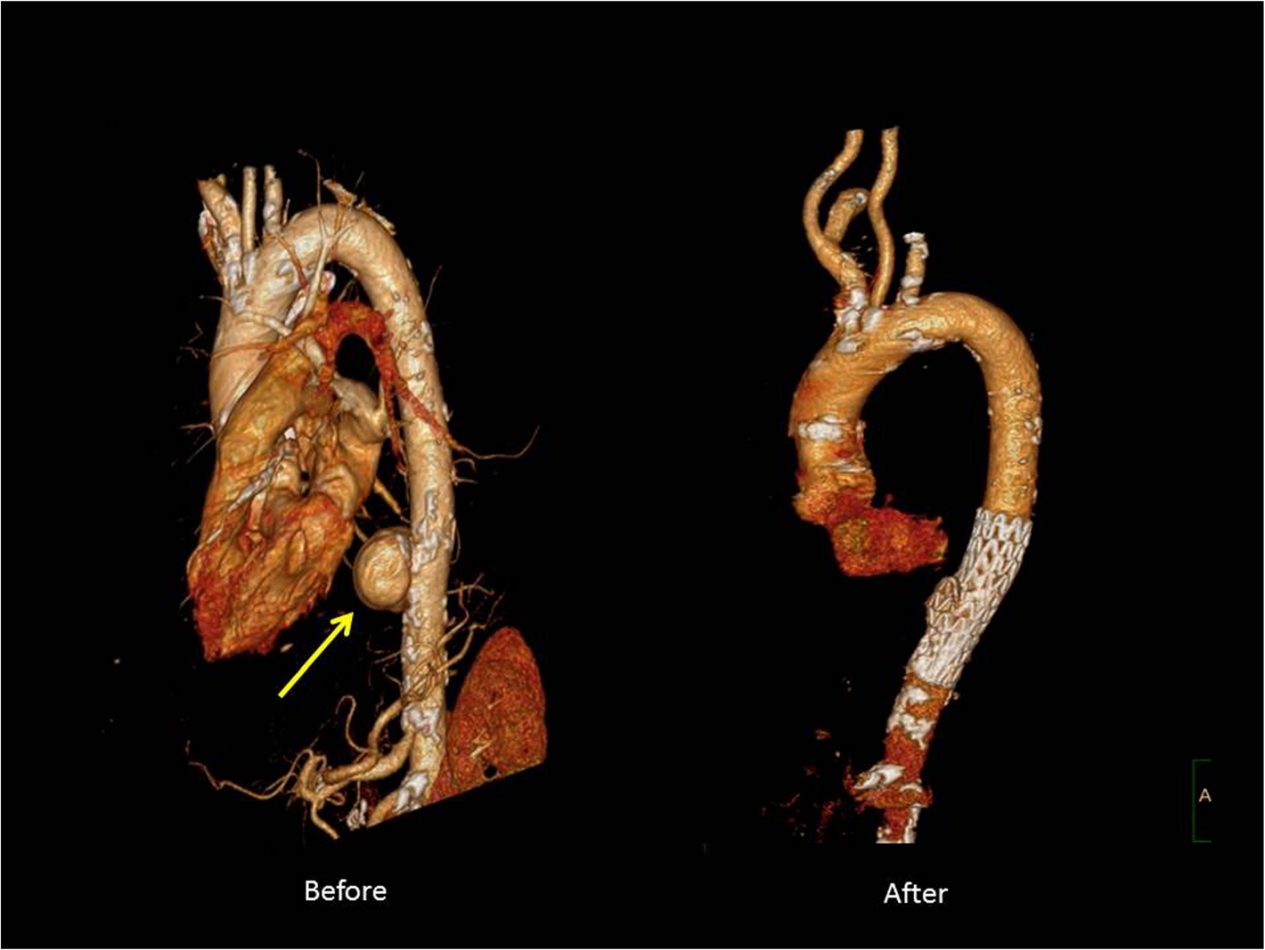
3D reconstruction of descending thoracic pseudoaneurysm caused by blunt aortic injury before (left) and after (repair) with TEVAR

**Fig. 2:**
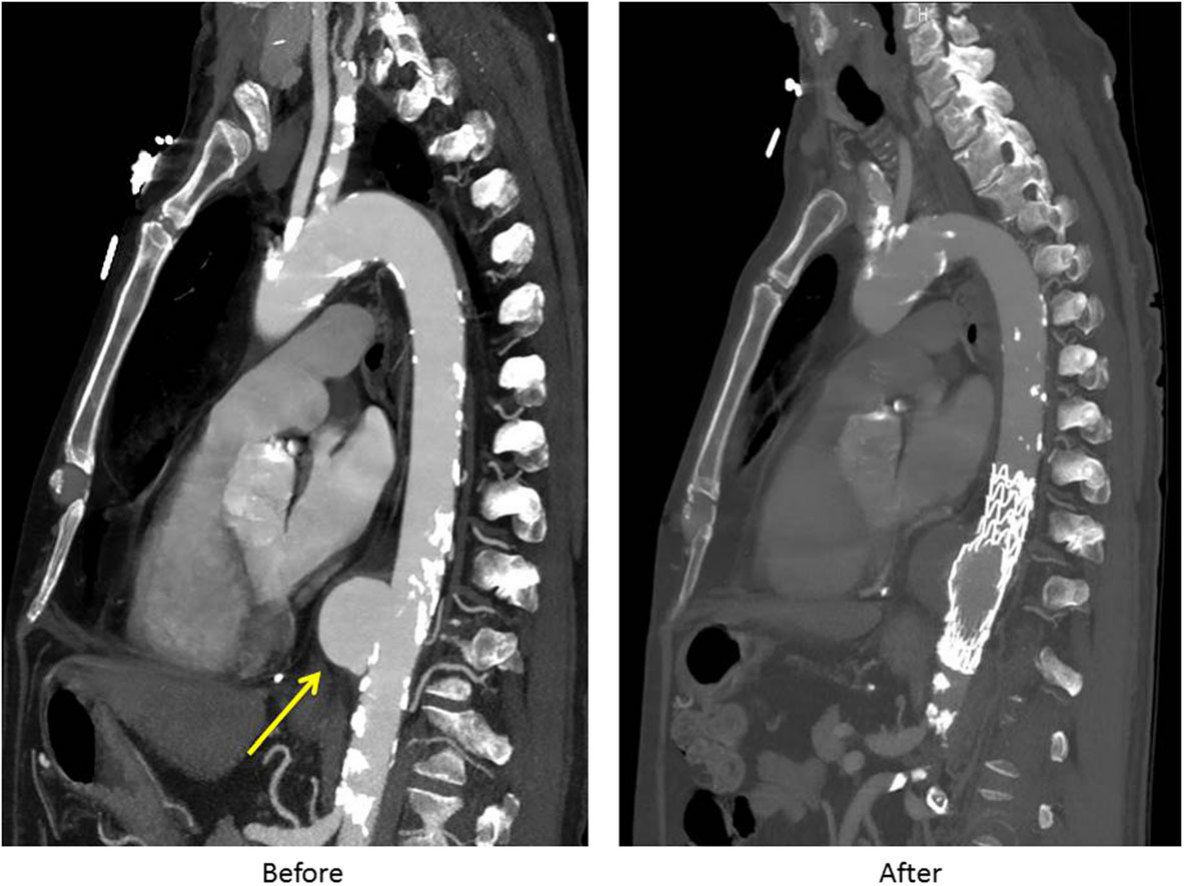
Computed tomography angiography in sagittal section of descending thoracic pseudoaneurysm caused by blunt aortic injury before (left) and after (repair) with TEVAR

Advantages of TEVAR are numerous. It obviates the need for open thoracotomy, single lung ventilation, aortic cross clamping and significant hemodynamic changes as well as the possibility of cardiopulmonary bypass. Furthermore, it is associated with shorter operative times as well as post-operative recovery (Figs. 1 and 2 demonstrate a repair of Grade III traumatic aortic pseudoaneurysm via TEVAR). A variety of retrospective series of patients with traumatic aortic injuries treated with TEVAR have demonstrated successful outcomes [47, 48]. Stoica and colleagues identified an early experience of 3 patients with acute rupture with successful TEVAR [48]. Pratesi and colleagues reported 11 patients treated with TEVAR successfully for acute aortic rupture with no neurologic deficits; the 30-day mortality was 9.1% [49]. Patel and colleagues reviewed 109 patients with 19 in the TEVAR cohort who had no early mortality or spinal cord ischemia [32].

TEVAR in the setting of blunt thoracic aortic injury however has some important limitations. Most notably, since most patients present with trauma, they tend to be younger in age. Tehrani et al. noted that oversizing may lead to endograft collapse [44]. The natural growth of the aorta in these younger patients may increase the possible risk for late device migration or endoleak. Furthermore, they will require longer surveillance of their endograft repair and the risks of radiation exposure may be problematic [19]. Even more so, sizing of the aorta in acute trauma with a patient that is hypovolemic with insufficient early resuscitation may lead to inaccuracy of aortic sizing which could lead to late endograft collapse and migration [32, 50]. Fortunately, meta-analyses and late outcome evaluations have noted satisfactory durability with acceptable complication and reintervention rates [32, 41, 47, 51, 52].

### Brief technique

For patient positioning, if no spinal fracture or other injuries contraindicate this, a roll is placed under the patients left chest to facilitate widening the arch. Also, the patient may be put on a bean bag in case a rapid open conversion is necessary. A wide prep and drape from bottom of the face to thighs bilaterally is recommended to allow for expedient trouble shooting in case open conversion or left carotid subclavian or other extra-anatomic bypasses are necessary.

Access can be obtained percutaneously or via open exposure. Once bilateral common femoral access is obtained, the ipsilateral limb is advanced after an appropriate sheath is inserted. Diagnostic imaging via a pigtail catheter can be placed via the contralateral femoral access or can be “stacked” on the ipsilateral side. Brachial access can also be used for the diagnostic catheter if necessary. Heparinization is an area of mild controversy and should be determined based on patient specific factors and the catalog of traumatic injuries. In the face of significant associated injuries, especially brain trauma, heparin can be omitted safely during the procedure. Using a glide wire and a catheter, the ascending aorta is cannulated from the ipsilateral side and true lumen is confirmed. Intravascular ultrasound (IVUS) is a helpful tool to ensure true lumen and should be used if available. Reentry devices are rarely necessary but should be available if needed. The catheter and wire are then exchanged for a stiff wire. Parallax should be corrected. Power injection may be performed with caution to keep the psi less than 500–600 to avoid rapid expansion of the injury and possible rupture. Once the device deployment landing zone is confirmed, several procedural vectors are reviewed. A high amount of torque may be developed during advancement of the device. This can be offset by advancing the graft past the intended deployment zone and then pulling the graft back to the intended site of deployment. This prevents the graft from malpositioning and shooting forward which may cover more aortic real estate than intended. The stiff wire can also be curled by the operator in the descending aorta into what is called a double curve. This can relieve some of the torque and avoid malpositioning. Prior to deployment, the blood pressure should be lowered to around 60 mm hg SBP to keep the blood pressure from pushing graft distal during deployment. Alternatively, rapid pacing may be employed. In addition, the pigtail is withdrawn distal to the stent graft deployment system. When the graft is deployed, the pigtail is re-advanced to the ascending aorta and a repeat image is performed to confirm placement. If no endoleak is noted, the graft is left alone. If persistent endoleak or positioning is questionable, compliant balloons are used to smooth out the graft and oppose the wall of the aorta better. Ballooning is avoided with aortic dissection pathology.

Arch vessels may need to be managed, such as the left subclavian artery, which may need to be covered partially or completely. In the acute setting of a young trauma patient, this is typically not an issue. If symptoms develop, a left carotid subclavian bypass may be required. If left common carotid artery coverage is required or occurs unintentionally, extra-anatomic bypass or open debranching may be required. An arterial line should be placed in the left arm during all these procedures. The arterial line waveform can be monitored during the procedure. This can correlate angiographically and help guide deployment. If the left subclavian artery is covered, the arterial line can also help determine the flow limiting consequence based on pre and post coverage changes in wave form analysis. A dominant left vertebral artery or a LIMA graft from a CABG may be best suited with a left carotid subclavian bypass prior to the procedure if partial or complete LSCA coverage is considered a possibility or planned; a vascular plug can be used to occlude the vessel to avoid retrograde endoleak. Transposition should not be performed as this may lead to no flow through the left vertebral artery or left internal mammary artery. Novel techniques have been described for left subclavian recanalization, including the use of laser atherectomy, rotational atherectomy and reentry devices to create a fenestration in the graft fabric coming retrograde from the left brachial artery. A snorkel or a chimney from the LSCA to the thoracic aorta can then be placed [53–55]. However, with the high patency rate of left carotid subclavian bypasses these techniques should be reserved for hostile necks and patients that may not tolerate the procedure; for instance, history of radiation, gross contamination (esophageal or tracheal injury), re-operative neck, tracheostomy, spit fistula, contralateral phrenic nerve injury, associated injuries of the neck that make the surgery less feasible, and others. The access sites are closed after the devices are removed. These patients should be kept in the intensive care unit and monitored for at least 24 h. At least 6 h of bedrest are required postoperatively and the access sites are monitored at appropriate time intervals.

### Complications

Infection, access site complications, endoleak, stent migration, stroke and paraplegia/paraparesis are all known but rare complications of TEVAR for blunt thoracic aortic injury with spinal cord injury being the most feared. Early complications are more common in open repair, but late complications are more common in endovascular repair. As noted previously, inappropriate sizing of the grafts can lead to migration, pseudo-coarctation, graft occlusion and endoleak. This may require reintervention or, rarely, open repair.

Long term surveillance is warranted because of potential late complications. It is common in the trauma population for patients to be more migratory and take on more financial/insurance related burdens. This can make long term follow up difficult [56]. Surveillance with CTA, MRA or TEE can be employed and they all have their pros and cons. Serially repeated CTAs in young trauma patients over the many years they will need surveillance predisposes them to the risks associated with long term repeat doses of ionizing radiation [19]. All common TEVAR stents are MRI safe and MRA can be employed later on in surveillance, but early imaging surveillance is typically preferred with CTA initially. TEE can be used when the other modalities are not desirable due to patient specific factors or when they are contraindicated. Some even use multiple view plain x ray to monitor the graft for migration and other issues while limiting radiation dosage [56].

Strokes postoperatively can be caused by embolization from wire, device and/or catheter manipulation in the aortic arch. It can also be caused by vertebrobasilar insufficiency caused by partial or complete coverage of the left subclavian artery. Left upper extremity ischemia, vertebrobasilar insufficiency and coronary steal are also rare complications that occur. Most patients with blunt thoracic aortic injury are young otherwise healthy patients that tolerate LSCA coverage with no issues [57, 58]. For instance, the RESCUE trial presented 52 patients who underwent TEVAR BTAI; 20 required complete LSCA coverage and 9 required partial LSCA coverage. Zero of these patients suffered any strokes or spinal cord injuries as a result and only 4 patients required LSCA revascularization [57]. If these complications arise, they can be treated with left carotid subclavian bypass.

## Conclusion

Blunt thoracic aortic injury requires a high index of suspicion based on mechanism of injury in the trauma population. Endovascular approaches have slowly replaced open surgical repair for the management of this pathology. Clearly, such patients that present with blunt thoracic injury should be relegated to centers that specialize in the polytrauma patient as it is their concurrent injuries that are the focus of their critical care.

## Data Availability

Not applicable.

## Abbreviations

ATLS: Advanced Trauma Life Support
CTA: Computed tomographic angiography
FAST: Focused Abdominal Sonography for Trauma
LIMA: Left internal mammary artery
LSCA: Left subclavian artery
MRA: Magnetic resonance angiography
OG/NG: Orogastric/Nasogastric
TEE: Transesophageal echocardiography
TEVAR: Thoracic endovascular aortic repair

## References

[1] Sevitt S (1977). The mechanism of traumatic rupture of the thoracic aorta. Brit J Surg.

[2] Cowley, RA, Turney SZ. Rupture of thoracic aorta caused by blunt trauma. A fifteen year experience J Thoracic Cardiovascular Surgery 1990. 100:652. 2232829

[3] Teixeira PG, Inaba K, Barmparas G (2011). Blunt thoracic aortic injuries: an autopsy study. J Trauma.

[4] de Mestral C, Dueck A, Sharma SS (2013). Evolution of the incidence, management, and mortality of blunt thoracic aortic injury: a population-based analysis. J Am Coll Surg.

[5] Chulman CI, Carvajal PL, PP. (2007). Incidence and crash mechanism of aortic injury during the past decade. J Trauma.

[6] Cannon, R. Jaimin, Trivedi. Pagni, S et al. Open repair of blunt thoracic aortic injury remains relevant in the endovascular era. J Am College Surg. 2012 943–949. 10.1016/j.jamcollsurg.2012.03.003 22541985

[7] Gaffey AC, Zhang J, Saka E, Quatromoni JG, Glaser J, Kim P, Szeto W, Kalapatapu V (2019). Natural history of nonoperative Management of Grade II blunt thoracic aortic injury. Ann Vasc Surg.

[8] Parmley LF, MattinglyTW,ManionWC, et al. Nonpenetrating traumatic injury of the aorta Circulation 1958;17:1086e101. 10.1161/01.cir.17.6.1086 13547374

[9] Crass JR, Cohen AM, Motta AO (1990). A proposed new mechanism of traumatic aortic rupture: the osseous pinch. Radiology.

[10] Mosquera V, Marini M, et al. Role of conservative management in traumatic aortic injury: comparison of long term results of conservative, surgical and endovascular treatment. J Thoracic Cardiovasc Surg. 2011:614–21. 10.1016/j.jtcvs.2010.10.044 21269644

[11] Feczko JD, Lynch L, Pless JE, Clark MA, McClain J, Hawley DA (1992). An autopsy case review of 142 non-penetrating (blunt) injuries of the aorta. J Trauma.

[12] Lundervall J (1964). The mechanism of traumatic rupture of the aorta. Acta Pathol Microbiolog Scandinavia.

[13] Crass, JR. Cohen AM. Motta AO. A proposed new mechanism of aortic rupture: the osseous pinch. Radiology 1990. 176:645. 10.1148/radiology.176.3.2389022 2389022

[14] Stemper. BD. Yogandandan N. Pintar FA. Brasej KJ. Multiple subfailures characterize blunt aortic injury. J Trauma 2007. 62:1171. 10.1097/TA.0b013e31804d4950 17495720

[15] Demetriades D, Velmahos GC, Scalea TM, Jurkovich GJ, Karmy-Jones R, Teixeira PG (2008). Diagnosis and treatment of blunt thoracic aortic injuries: changing perspectives. J Trauma.

[16] Malhotra AK, Fabian TC, Croce MA (2001). Minimal aortic injury: a lesion associated with advancing diagnostic techniques. J Trauma.

[17] Pate JW, Fabian TC, Walker W (1995). Traumatic rupture of the aortic isthmus: an emergency?. World J Surg.

[18] Fabian TC, Davis KA, Gavant ML, Croce MA, Melton SM, Patton JH (1998). Prospective study of blunt aortic injury and helical CT is diagnostic and antihypertensive therapy reduces rupture. Am Surg.

[19] Azizzadeh A, Keyhani K, Miller CC (2009). Blunt traumatic aortic injury: initial experience with endovascular repair. J Vasc Surg.

[20] Lee WA, Matsumura JS, Mitchell RS (2011). Endovascular repair of traumatic thoracic aortic injury: clinical practice guidelines of the society for vascular surgery. J Vasc Surg.

[21] Akins CW, Buckley MJ, Daggett W, McIlduff JB, Austin WG (1981). Acute traumatic disruption of the thoracic aorta: a ten year experience. Ann Thorac Surg.

[22] Pate JW, Gavant ML, Weiman DS, Fabian TC (1999). Traumatic rupture of the aortic isthmus: program of selective management. World J Surg.

[23] Hemmila MR, Arbabi S, Rowe SA, Brandt M, Wang SC, Taheri PA (2004). Delayed repair for blunt thoracic aortic injury: is it really equivalent to early repair. J Trauma.

[24] Demetriades D, Velmahos GC, Scalea TM, Jurkovich GJ, Karmy-Jones R, Teixeira PG (2009). Blunt traumatic thoracic aortic injuries: early or delayed repair—results of an American Association for the Surgery of Trauma prospective study. J Trauma.

[25] Williams JS, Graff JA, Uku JM, Steinig. Aortic injury in vehicular trauma. Ann Thorac Surg 1994;57:726–730. 10.1016/0003-4975(94)90576-2 8147647

[26] Eddy AC, Rusch VW, Fligner CL, Reay DT, Rice CL (1990). The epidemiology of traumatic rupture of the thoracic aorta in children: a thirteen year review. J Trauma.

[27] Cowley RA, Turney SZ, Hankins JR, Rodriguez A, Attar S, Shankar BS (1990). Rupture of thoracic aorta caused by blunt trauma: a fifteen-year experience. J Thorac Cardiovasc Surg.

[28] von Oppell UO, Dunne TT, De Groot MK, Zilla P (1994). Traumatic aortic rupture: twenty-year meta-analysis of mortality and risk for paraplegia. Ann Thorac Surg.

[29] Amabile P, Collart F, Gariboldi V, et al. Surgical versus endovascular treatment of traumatic thoracic aortic rupture. SVS. Nov 2004:873–9. 10.1016/j.jvs.2004.08.053 15557899

[30] Dake MD, Miller DC, Semba CP, Mitchell RS, Walker PJ, Liddell RP (1994). Transluminal placement of endovascular stent-grafts for the treatment of descending thoracic aortic aneurysms. N Engl J Med.

[31] Semba CP, Kato N, Kee ST, Lee GK, Mitchell RS, Miller DC, Dake MD (1997). Acute rupture of the descending thoracic aorta: repair with use of endovascular stent-grafts. J Vasc Interv Radiol.

[32] Patel HJ, Hemmila MR, Williams DM, Diener AC, Deeb GM (2011). Late outcomes following open and endovascular repair of blunt thoracic aortic injury. J Vasc Surg.

[33] Kato N, Dake MD, Miller DC, Semba CP, Mitchell RS, Razavi MK (1997). Traumatic thoracic aortic aneurysm: treatment with endovascular stent-grafts. Radiology.

[34] Demetriades D, Velmahos GC, Scalea TM, Jurkovich GJ, Karmy-Jones R, Teixeira PG (2008). Operative repair or endovascular stent graft in blunt traumatic thoracic aortic injuries: results of an American Association for the Surgery of Trauma prospective study. J Trauma.

[35] Takagi H, Kawai N, Umemoto T (2008). A meta-analysis of comparative studies of endovascular vs. open repair for blunt thoracic aortic injury. J Thorac Cardiovasc Surg.

[36] Patel HJ, Williams DM, Upchurch GR, Shillingford MS, Dasika NL, Proctor MC, Deeb GM (2006). Long term results from a 12-year experience with endovascular therapy for thoracic aortic disease. Ann Thorac Surg.

[37] Czermak BV, Waldenberger P, Fraedrich G, Dessl AH, Roberts K, Bale RJ (2000). Treatment of Stanford type B aortic dissection with stentgrafts: preliminary results. Radiology.

[38] Nienaber CA, Fattori R, Lund G, Dieckman C, Wolf W, Nicolas V (1999). Nonsurgical reconstruction of thoracic aortic dissection by stent-graft placement. N Engl J Med.

[39] Murgo S, Dussaussois L, Golzarian J, Cavenaile JC, Abada HT, Ferreira J (1998). Penetrating atherosclerotic ulcer of the descending thoracic aorta: treatment by endovascular stent-graft. Cardiovasc Interv Radiol.

[40] Dake MD, Kato N, Mitchell RS, Semba CP, Razavi MK, Shimono T (1999). Endovascular stent-graft placement for the treatment of acute aortic dissection. N Engl J Med.

[41] Xenos ES, Abedi NN, Davenport DL, Minion DJ, Hamdallah O, Sorial EE (2008). Meta-analysis of endovascular vs open repair for traumatic descending thoracic aortic rupture. J Vasc Surg.

[42] Galli R, Pacini D, Di Bartolomeo R, Fattori R, Turinetto B, Grillone G (1998). Surgical indications and timing of repair of traumatic ruptures of the thoracic aorta. Ann Thorac Surg.

[43] Pierangeli A, Turinetto B, Galli R, Caldarera L, Fattori R, Gavelli G (2000). Delayed treatment of isthmic aortic rupture. Cardiovasc Surg.

[44] Tehrani HY, Peterson BG, Katariya K, Morasch MD, Stevens R, DiLuozzo G (2006). Endovascular repair of thoracic aortic tears. Ann Thorac Surg.

[45] Jahromi AS, Kazemi K, Safar HA, Doobay B, Cina CS (2001). Traumatic rupture of the thoracic aorta: cohort study and systematic review. J Vasc Surg.

[46] Fabian TC, Richardson JD, Croce MA, Smith JS, Rodman J, Kearney PA (1997). Prospective study of blunt aortic injury: multicenter trial of the American Association for the Surgery of Trauma. J Trauma.

[47] Dunham MB, Zygun D, Petrasek P, Kortbeek JB, Karmy-Jones R, Moore RD (2004). Endovascular stent grafts for acute blunt aortic injury. J Trauma.

[48] Stoica L, Chocron S, Falcoz P, Etivent JP (2003). Endovascular stent grafting for contained rupture of the descending thoracic aorta. Eur J Cardiothor Surg.

[49] Pratesi C, Dorigo W, Troisi N, Pratesi G, Santoro G, Stefano P (2006). Acute traumatic rupture of the descending thoracic aorta: endovascular treatment. Am J Surg.

[50] Joker FHW, Verhagen HJM, Mojibian H, Davis KA, Moll FL, Muhs BE (2010). Aortic endograft sizing in trauma patients with hemodynamic instability. J Vasc Surg.

[51] Stampfl P, Greitbauer M, Zimpfer D, Fleck T, Schoder M, Lammer J (2006). Mid-term results of conservative, conventional and endovascular treatment for acute traumatic aortic lesions. Eur J Vasc Endovasc Surg.

[52] Pacini D, Angeli E, Fattori RL, Rocchi G, Marco LD (2005). Traumatic rupture of the thoracic aorta: ten years of delayed management. J Thorac Cardiovasc Surg.

[53] Bradshaw RJ, Ahanchi SS, Powell O, Larion S, Brandt C, Soult MC, Pannelton JM (2017). Left subclavian artery revascularization in zone 2 thoracic endovascular aortic repair is associated with lower stroke risk across all aortic disease. J Vasc Surgery.

[54] Tan TW, Coulter AH, Zhang WW (2016). Percutaneous in situ left subclavian artery fenestration using reentry catheter during endovascular thoracic aortic aneurysm repair. Int J angio.

[55] Redlinger RE, Ahnanchi SS, Panneton JM (2013). In situ laser fenestration during emergent thoracic endovascular aortic repair is an effective method for left subclavian artery revascularization. J Vasc Surg.

[56] Steuer J, Bjorck M, Sonesson B, Resch T, Dias N, Hultgren R (2015). Editor's choice – durability of endovascular repair in blunt traumatic thoracic aortic injury: long-term outcome from four tertiary referral centers. Eur J Vasc Endovasc Surg.

[57] Khozynezhad A. Donayre CA. Azizzadeh A. White R. RESCUE. One year results of thoracic endovascular aortic repair for blunt thoracic aortic injury (RESCUE trial. J Thorac Cardiovasc Surg 2015 149 (1) 155–161. 10.1016/j.jtcvs.2014.09.026 25439771

[58] Serra, Rafaele. Franciscis S. et al. Endovascular repair for acute traumatic transection of the descending thoracic aorta: experience of a single centre with a 12 years follow up. J Cardiothoracic Surg. 2015. 10:171. 10.1186/s13019-015-0388-5 PMC4655082 26590963

